# Epidemiology, Management, and Outcomes of Patients Hospitalized With Community-Acquired Infection in a Resource-Limited Setting in Southeast Asia: A Prospective Observational Study

**DOI:** 10.1093/ofid/ofag022

**Published:** 2026-01-14

**Authors:** Rungnapa Phunpang, Prapassorn Poolchanuan, Taylor D Coston, Adul Dulsuk, Sopha Saeyang, Boonthanom Moonmueangsan, Narongchai Sangsa, Sermchart Chinnakarnsawas, Rachan Janon, T Eoin West, Narisara Chantratita, Shelton W Wright

**Affiliations:** Department of Microbiology and Immunology, Faculty of Tropical Medicine, Mahidol University, Bangkok, Thailand; Department of Microbiology and Immunology, Faculty of Tropical Medicine, Mahidol University, Bangkok, Thailand; Division of Pulmonary, Critical Care and Sleep Medicine, Department of Medicine, University of Washington, Seattle, Washington, USA; Department of Microbiology and Immunology, Faculty of Tropical Medicine, Mahidol University, Bangkok, Thailand; Department of Microbiology and Immunology, Faculty of Tropical Medicine, Mahidol University, Bangkok, Thailand; Department of Microbiology and Immunology, Faculty of Tropical Medicine, Mahidol University, Bangkok, Thailand; Department of Medicine, Roi Et Hospital, Roi Et, Thailand; Department of Medicine, Roi Et Hospital, Roi Et, Thailand; Department of Medicine, Mukdahan Hospital, Mukdahan, Thailand; Division of Pulmonary, Critical Care and Sleep Medicine, Department of Medicine, University of Washington, Seattle, Washington, USA; Department of Global Health, University of Washington, Seattle, Washington, USA; Department of Microbiology and Immunology, Faculty of Tropical Medicine, Mahidol University, Bangkok, Thailand; Mahidol-Oxford Tropical Medicine Research Unit, Faculty of Tropical Medicine, Mahidol University, Bangkok, Thailand; Division of Pediatric Critical Care Medicine, Department of Pediatrics, University of Washington, Seattle, Washington, USA

**Keywords:** AKI, global infection, resource limited, sepsis, tropical infection

## Abstract

**Background:**

In many resource-limited settings, hospitalization for community-acquired infection is common, but data regarding illness severity, etiology, and morbidity remain sparse.

**Methods:**

We conducted a prospective observational study from May 2022 to August 2023 at 2 hospitals in northeast Thailand. Adults hospitalized with community-acquired infection were enrolled within 24 hours of admission and followed up to 28 days. We identified patients meeting sepsis criteria and assessed related epidemiology, management, and mortality risk factors.

**Results:**

Of 1445 patients screened, 940 were enrolled. The median age was 60 years and preexisting diabetes mellitus was common (42%). Sixty-six percent of patients met sepsis criteria. Blood cultures and broad-spectrum antibiotics on admission were common (both >95%), although lactate measurement was performed in 43% of patients with sepsis. In patients with sepsis, critical illness outside the intensive care unit was common on medical ward admission, including respiratory failure (33%) and shock (21%). Tropical etiologies of infection included melioidosis (8%) and leptospirosis (4%), and gram-negative organisms accounted for 81% of bacteremia. Twenty percent of patients with sepsis died by 28 days. Sepsis-associated acute kidney injury (SA-AKI) on admission was independently associated with mortality (adjusted odds ratio, 2.07; 95% CI, 1.30–3.29; *P* = .002), and patients with SA-AKI had worse survival (*P* < .001) than those without.

**Conclusions:**

In rural Southeast Asia, sepsis is common among patients hospitalized with infection and associated with substantial morbidity and mortality. Distinct pathogens and broad-spectrum antibiotics are common, even in the absence of sepsis. We identified several modifiable risk factors of death, including SA-AKI, potentially influencing initial management in similar settings.

Sepsis disproportionally affects low- and middle-income countries (LMICs), with millions of estimated deaths annually [1]. In tropical regions of Southeast Asia, sepsis-related morbidity is particularly high and caused by a diverse group of pathogens [2]. The heterogeneity and global variation in this disease therefore necessitate further study to develop contextualized and comprehensive management plans [3].

The Third International Consensus Definition for Sepsis defined sepsis as infection-related host response dysregulation leading to organ dysfunction [4]. Implicit in this definition were measures of organ dysfunction typically requiring critical care interventions. As such, many studies of sepsis have focused on patients in the intensive care unit (ICU), even though patients with sepsis may be identified outside of such settings [5]. Furthermore, in resource-limited settings, patients frequently do not have access to critical care, or they receive critical care outside an ICU, requiring modifications of traditional management approaches [6, 7].

Studies of patients hospitalized with community-acquired infection are limited in Southeast Asia, despite its high estimated burden and unique tropical etiologies. Several regional studies of patients with suspected or confirmed sepsis in large academic or referral centers may miss broader populations of patients who are infected [2, 8]. Therefore, in this study, we sought to identify the frequency of sepsis among a large, prospectively enrolled cohort of patients admitted for community-acquired infection at 2 rural hospitals in northeast Thailand. We then assessed the causative infectious etiologies, management, risk factors, and infection-related outcomes in this unique and understudied population.

## METHODS

### Study Design, Participants, and Outcomes

We conducted a prospective observational study at 2 hospitals in northeastern Thailand from May 2022 through August 2023. Patients hospitalized at Mukdahan Hospital, Mukdahan province, Thailand, and Roi Et Hospital, Roi Et province, Thailand, were screened within 24 hours of hospital admission to determine if they met enrollment criteria of age ≥18 years and a primary admission diagnosis of suspected or confirmed infection. Exclusion criteria, regional and site information, and additional study design are available in the supplemental methods. Enrolled patients were then subsequently followed during their hospitalization. All patients, including postdischarge, were followed to or contacted at 28 days following enrollment to determine death after discharge. Discharged patients not contacted were not included in analyses related to 28-day mortality.

### Definitions

Sepsis was defined in accordance with the Third International Consensus Definition for Sepsis [4]. A Sequential Organ Failure Assessment (SOFA) score was calculated by parameters within the first 24 hours of admission, and sepsis was defined as patients with suspected infection and a SOFA score ≥2. Modifications to the respiratory component of the SOFA score were necessary as arterial blood gas measurement is uncommon. For patients without an available partial pressure of arterial oxygen (PaO_2_), a ratio of the pulse-oximetric oxygen saturation to the fraction of inspired oxygen (SpO_2_/FiO_2_) was calculated for patients with an SpO_2_ <98% [9, 10]. As previously reported, including adoption in resource-constrained settings, SpO_2_/FiO_2_ equivalents for the SOFA PaO_2_/FiO_2_ cutoffs were calculated by the following equation: SpO_2_/FiO_2_ = 64 + [0.84 × (PaO_2_/FiO_2_)] [11, 12]. Otherwise, patients who were intubated or mechanically ventilated with SpO_2_ >98% and no available PaO_2_ were given a default respiratory SOFA score of 2 out of 4 (Supplementary Table 1) [8]. A Glasgow Coma Scale score was recorded at enrollment by the study team. To maintain consistency with prior sepsis investigations, when score components were not available (Supplementary Table 2), they were assumed to be normal [1, 13]. The dates of receipt of antibiotics, blood cultures, and lactate were available but not the minute/hour. Therefore, timing of sepsis bundles was limited to the calendar day of admission.

Acute kidney injury (AKI) was determined by criteria from the 2012 Kidney Disease: Improving Global Outcomes guidelines [14]. AKI was defined as a creatinine value within 24 hours of admission ≥150% of a baseline creatinine. If a baseline creatinine value was not available, an estimated value was calculated in patients without known chronic kidney disease per the “modification of diet in renal disease” equation, with an estimated glomerular filtration rate of 75 mL/min/1.73 m^2^. In all patients not requiring baseline peritoneal dialysis or hemodialysis, an increase in creatinine by 0.3 mg/dL during the first 48 hours of admission was also classified as AKI. Urine output was not available and so not included in the AKI definition. Additional definitions related to critical illness, presenting clinical syndromes, and infectious disease diagnoses can be found in the supplemental methods.

### Statistical Analyses

Data were summarized by proportions for discrete variables and median (IQR) for continuous variables. Differences in proportions and medians between groups were assessed by χ^2^ and the Mann-Whitney test, respectively. To identify risk factors of 28-day mortality, 44 clinical characteristics available at enrollment were a priori identified. In univariate analyses, the association of each variable with 28-day mortality was calculated by logistic regression. To identify a set of variables for inclusion in multivariable models, all 44 clinical variables were subjected to logistic regression analysis by least absolute shrinkage and selection operator (LASSO) methodology, in which lambda was selected by the bayesian information criterion. The selected risk factors were confirmed with the lambda that minimizes the minimal mean squared prediction error [15]. The LASSO-identified risk factors were then assessed for their association with 28-day mortality in multivariable models, which included enrollment site. Curves for 28-day survival were compared via the log-rank test. *P* values <.05 were considered significant. Analyses were performed in Stata/SE version 14.2.

### Patient Consent Statement

Written informed consent was obtained from all study participants or their surrogate decision makers. The study was approved by the ethics committees of Mukdahan Hospital (MEC 09/64), Roi Et Hospital (RE035/2564), the Mahidol University Faculty of Tropical Medicine (MUTM 2021-043-01), and the University of Washington (STUDY00012758).

## RESULTS

### Cohort Characteristics

Of 1445 patients screened, 940 were enrolled and included in analyses (Supplementary Figure 1). Enrolled patients were predominantly male (551/940, 59%) with a median age of 60 years (IQR, 49–70; Table 1, Supplementary Table 3). Notably, diabetes mellitus was common (396/940, 42%), and 44% (416/940) of patients were referred from another facility, typically on the same day as initial presentation. A total of 618 (66%) patients met sepsis criteria within 24 hours of admission. Patients with sepsis at enrollment tended to have more comorbidities vs those without sepsis by Charlson Comorbidity Index (*P* < .001), although diabetes mellitus was more common in patients without sepsis (*P* = .02). Patients with sepsis were more likely to have an admission diagnosis of pneumonia (272/618, 44%) than those without sepsis (82/322, 26%; *P* < .001). Conversely, patients with sepsis were less likely to have an admission diagnosis of an acute febrile illness (sepsis, 110/618, 18%; nonsepsis, 94/322, 29%; *P* < .001).

**Table 1.** Characteristics of Patients With Community-Acquired Infection

	Median (IQR) or No. (%)			
Characteristics	Entire Cohort (N = 940)	Sepsis ^a^ (n = 618)	Nonsepsis (n = 322)	*P* Value
Demographics				
Age, y	60 (49–70)	61 (50–72)	57 (46–68)	<.001
Female sex	389 (41)	233 (38)	156 (49)	<.01
Rainy season presentation	614 (65)	390 (63)	224 (70)	.05
Preexisting conditions				
Charlson Comorbidity Index	2 (1–4)	3 (1–4)	2 (1–3)	<.001
Diabetes mellitus	396 (42)	243 (39)	153 (48)	.02
Hypertension	356 (38)	240 (38)	116 (36)	.40
Chronic kidney disease	139 (15)	115 (19)	24 (8)	<.001
Dyslipidemia	97 (10)	60 (10)	37 (12)	.39
Stroke	60 (6)	45 (7)	15 (5)	.12
Chronic lung disease	55 (6)	34 (6)	20 (6)	.73
Chronic cardiovascular disease	41 (4)	36 (6)	5 (2)	<.01
Chronic steroid use	34 (4)	22 (4)	12 (4)	.90
Cancer	24 (3)	17 (3)	7 (2)	.59
Chronic liver disease	24 (3)	24 (4)	0	<.001
HIV	17 (2)	12 (2)	5 (2)	.68
Rheumatologic disorders	16 (2)	12 (2)	4 (1)	.43
Hospitalization				
Referral from another facility	416 (44)	350 (57)	66 (21)	<.001
Days to referral	0 (0–0)	0 (0–0)	0 (0–0)	.93
Admission ward				
Medical	810 (86)	509 (82)	301 (93)	<.001
Surgical	28 (3)	9 (2)	19 (6)	
Intensive care unit	102 (11)	100 (16)	2 (1)	
Length of hospitalization, d	6 (3–13)	6 (3–13)	5 (3–11)	<.001
Procedural drainage-debridement	87 (9)	56 (9)	31 (10)	.78
Presenting clinical syndromes ^b^				
Pneumonia	354 (38)	272 (44)	82 (26)	<.001
Acute febrile illness	204 (22)	110 (18)	94 (29)	<.001
Gastrointestinal illness	132 (14)	81 (13)	51 (16)	.25
Skin or soft tissue infection	27 (3)	14 (2)	13 (4)	.12
Urinary tract infection	73 (8)	48 (8)	25 (8)	.99
Intracranial infection	4 (0)	3 (1)	1 (0)	.7
Abscess	33 (4)	14 (2)	19 (6)	<.01

^a^Sepsis defined as modified Sequential Organ Failure Assessment score ≥2 in patients with community-acquired infection.

^b^Presenting clinical syndromes based on primary admission diagnosis.

### Initial Management and Outcomes by Sepsis Classification

On admission to the study hospitals, blood cultures were sent on 96% of patients (902/940; Table 2, Supplementary Table 4). Antibiotics were frequently given on admission (917/940, 98%) and overwhelmingly included broad gram-negative coverage (912/940, 97%). Ceftazidime and ceftriaxone were the most frequently received antibiotics at the study hospitals or referring sites (Supplementary Table 5). Antibiotics started on admission were continued for a median 6 days (sepsis, 6 days [IQR, 3–13]; nonsepsis, 4 days [IQR, 3–11]; *P* = .002). Finally, lactate was measured for 32% (302/940) of patients on admission and was more frequently measured for patients with sepsis as opposed to those without (264/618 [43%] vs 38/322 [12%]; *P* < .001). Given the inconsistency of lactate measurement, adherence to the recommended sepsis bundle on admission (blood cultures, antibiotics, and lactate measurement) per the 2021 Surviving Sepsis Campaign (SSC) occurred in only 41% of patients with sepsis (255/618) [16].

**Table 2.** Management, Severity of Illness, and Outcomes of Patients With Community-Acquired Infection

	No. (%) or Median (IQR)			
Characteristic	Entire Cohort (N = 940)	Sepsis (n = 618)	Nonsepsis (n = 322)	*P* Value
Admission management				
Blood cultures sent on admission	902 (96)	596 (96)	306 (95)	.30
Antibiotics received on admission	917 (98)	604 (98)	313 (97)	.62
Broad gram-negative coverage ^a^	912 (97)	603 (98)	309 (96)	.17
MRSA coverage ^b^	185 (20)	126 (20)	59 (18)	.45
Duration, d	6 (3–12)	6 (3–13)	4 (3–11)	.002
Lactate measured on admission	302 (32)	264 (43)	38 (12)	<.001
Admission illness characteristics				
SOFA	3 (1–7)	5 (3–9)	0 (0–1)	<.001
qSOFA	1 (1–2)	2 (1–2)	1 (0–1)	<.001
Acute kidney injury ^c^	261 (28)	239 (39)	22 (7)	<.001
Outcomes during hospitalization				
Mechanical ventilation	322 (34)	315 (51)	7 (2)	<.001
Vasoactive medication	275 (29)	267 (43)	8 (3)	<.001
New kidney replacement therapy	44 (5)	43 (7)	1 (0)	<.001
Intermittent hemodialysis	44 (5)	43 (7)	1 (0)	<.001
Peritoneal dialysis	0	0	0	
Mortality outcomes				
7 d	78 (8)	75 (12)	3 (1)	<.001
Days to death	2 (1–5)	2 (1–5)	5 (1–6)	.35
28 d	132 (14)	125 (20)	7 (2)	<.001
Days to death	6 (2–13)	6 (2–12)	20 (6–23)	.10
Lost to 28-d follow-up	6 (1)	4 (1)	2 (1)	>.99

Abbreviations: KDIGO, Kidney Disease: Improving Global Outcomes; MRSA, methicillin-resistant *Staphylococcus aureus*; qSOFA, quick Sequential Organ Failure Assessment; modified SOFA, Sequential Organ Failure Assessment.

^a^Gram-negative coverage on or prior to admission, including receipt of a third- or fourth-generation cephalosporin, carbapenem, piperacillin-tazobactam, or levofloxacin.

^b^Community-acquired MRSA coverage on or prior to admission, including receipt of vancomycin, doxycycline, or clindamycin.

^c^Acute kidney injury based on KDIGO definition of 150% of estimated baseline creatinine in patients without chronic kidney disease or increase in creatinine by 0.3 mg/dL within 48 hours in any patient.

A total of 132 patients (14%) died within 28 days after enrollment (Table 2, Supplementary Table 4). Highlighting local cultural practices, 67 (51%) of those who died by 28 days were discharged from the hospital prior to their death, and 41 (62%) died within 1 day of discharge. Death within 28 days of enrollment was more common in patients with sepsis (125/618, 20%) than those without sepsis (7/322, 2%; *P* < .001). Out of 940 enrolled patients, 6 were lost to follow-up at 28 days after enrollment.

### Infectious Diseases by Sepsis Classification

We next characterized the infectious diseases represented in the cohort (Table 3, Supplementary Table 6). Overall, 18% (164/940) had a positive blood culture from admission. Bacteremia was more common in patients with sepsis (130/618, 21%) than those without sepsis (34/322, 10%; *P* < .001). Gram-negative bacteria accounted for most positive blood cultures (133/164, 81%), including patients with sepsis (102/130, 78%) and without (31/34, 91%). *Burkholderia pseudomallei*, the antibiotic-resistant bacteria endemic in northeast Thailand and the cause of the severe tropical infection melioidosis, was the most common bacteria identified in blood cultures. We compared other tropical infectious diseases based on final hospital diagnosis. Leptospirosis was diagnosed in 36 of 940 (4%) patients, with 92% (33/36) meeting sepsis criteria. Other infectious diseases were diagnosed in the cohort, such as acute tuberculosis (24/940, 3%), scrub typhus (14/940, 2%), dengue (7/940, 1%), and nonbacteremic melioidosis (20/940, 2%). Notably, no specific bacteremia pathogen differed by sepsis category, and the majority of the cohort did not have a specific infectious etiology identified.

**Table 3.** Infectious Diseases in Patients With Community-Acquired Infection

Characteristic	Entire Cohort (N = 940)	Sepsis (n = 618)	Nonsepsis (n = 322)	*P* Value
Bloodstream infection	164 (18)	130 (21)	34 (10)	<.001
Gram-negative bacteremia	133 (14)	102 (17)	31 (10)	.004
*Burkholderia pseudomallei*	58 (6)	43 (7)	15 (5)	.17
*Escherichia coli*	39 (4)	27 (4)	12 (4)	.64
*Klebsiella* spp	21 (2)	16 (3)	5 (2)	.37
*Pseudomonas* spp	3 (0)	3 (1)	0	.56
*Acinetobacter baumannii*	5 (1)	5 (1)	0	.17
*Salmonella* serogroup D	5 (1)	5 (1)	0	.17
*Citrobacter koseri*	2 (0)	2 (0)	0	.55
*Vibrio parahemolyticus*	1 (0)	1 (0)	0	>.99
*Aeromonas* spp	1 (0)	1 (0)	0	>.99
Gram-positive bacteremia	32 (3)	29 (5)	3 (1)	.002
*Staphylococcus aureus*	11 (1)	10 (2)	1 (0)	.11
*Streptococcus pneumoniae*	4 (0)	4 (1)	0	.31
*Streptococcus pyogenes*	6 (1)	5 (1)	1 (0)	.67
Group G *Streptococcus*	3 (0)	3 (1)	0	.56
Group D *Streptococcus*	2 (0)	2 (0)	0	.55
Other *Streptococcus* spp	2 (0)	2 (0)	0	.55
*Enterococcus faecalis*	4 (0)	3 (1)	1 (0)	.67
Polymicrobial	5 (1)	4 (1)	1 (0)	>.99
Contaminant organisms ^a^	35 (4)	28 (5)	7 (2)	.07
Other infectious etiologies ^b^				
Leptospirosis	36 (4)	33 (5)	3 (1)	<.001
Acute tuberculosis disease	24 (3)	10 (2)	14 (4)	.02
Nonbacteremic melioidosis	20 (2)	12 (2)	8 (3)	.64
Scrub typhus	14 (2)	12 (2)	2 (1)	.16
Dengue	7 (1)	4 (1)	3 (1)	.70
Malaria	1 (0)	1 (0)	0	>.99

^a^Contaminant organisms include coagulase-negative *Staphylococcus* spp, alpha-hemolytic *Streptococcus* spp, *Propionibacterium* spp, *Corynebacterium* spp, *Burkholderia cepacia*, or *Bacillus* spp if no other clinical evidence existed suggestive of infection.

^b^Listed infectious etiologies were based on final diagnoses according to local hospital testing and diagnostic protocols. Nonbacteremic melioidosis diagnoses were based on positivity of any nonblood culture for *B pseudomallei*.

### Critical Illness in Sepsis

As many patients in resource-limited settings receive medical care outside of traditional ICUs, we next sought to characterize the medical management of patients with sepsis inside and outside the ICU (Table 4). In 618 patients with sepsis, 176 (28%) were initially admitted to an ICU while 444 (72%) were admitted to a ward. Notably, critical care was still common in patients with sepsis outside the ICU, as 27% (121/444) required mechanical ventilation and 21% (92/444) required vasoactive medications within 24 hours of admission. Patients with sepsis initially admitted to the ICU had higher 28-day mortality (34%) and frequency of AKI on admission (54%) as compared with those with critical illness but admitted to the medical ward (19% [*P* = .001] and 38% [*P* = .002], respectively; Supplementary Table 7).

**Table 4.** Clinical Management and Outcomes of Patients With Sepsis by Admission Ward

	Median (IQR) or No. (%)			
Characteristic	All Sepsis (n = 618)	ICU (n = 174)	Ward (n = 444)	*P* Value
Severity of illness at enrollment				
SOFA	5 (3–9)	10 (7–13)	4 (3–6)	<.001
APACHE II	…	22 (17–25)	…	…
Acute kidney injury ^a^	239 (39)	94 (53)	145 (33)	<.001
Critical care at enrollment				
Respiratory failure ^b^	306 (50)	159 (90)	147 (33)	<.001
Mechanical ventilation	277 (45)	156 (89)	121 (27)	<.001
Vasoactive medications	198 (32)	106 (60)	92 (21)	<.001
New kidney replacement therapy	15 (3)	13 (7)	2 (1)	<.001
Admission management				
Blood cultures sent	596 (96)	172 (99)	424 (96)	.05
Antibiotics received	604 (98)	172 (99)	432 (97)	.37
Procedural drainage/debridement	56 (9)	16 (9)	40 (9)	.94
Lactate concentration measured	264 (43)	96 (55)	168 (38)	<.001
Death				
7 d	75 (12)	38 (22)	37 (8)	<.001
Days to death	2 (1–5)	2 (1–5)	3 (1–5)	.48
28 d	125 (20)	60 (34)	65 (15)	<.001
Days to death	6 (2–12)	5 (1–12)	6 (2–13)	.34
Lost to 28-d follow-up	4 (1)	0	4 (1)	.58

Abbreviations: ICU, intensive care unit; KDIGO, Kidney Disease: Improving Global Outcomes; modified SOFA, Sequential Organ Failure Assessment.

^a^Acute kidney injury based on KDIGO definition of 150% of estimated baseline creatinine in patients without chronic kidney disease or increase in creatinine by 0.3 mg/dL within 48 hours in any patient not requiring dialysis at baseline.

^b^Respiratory failure defined as requiring endotracheal intubation, mechanical ventilation, or noninvasive positive pressure ventilation.

### Risk Factors of 28-Day Mortality in Sepsis

**Figure 1.**
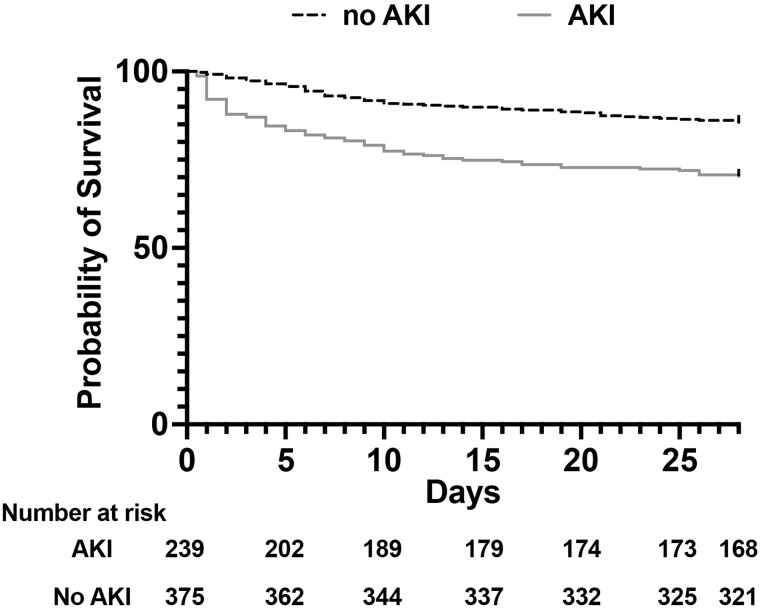
Survival curve of sepsis cases with and without AKI. Kaplan-Meier survival curves over 28 days demonstrate worse survival in patients with sepsis-associated AKI (gray) at enrollment vs those without AKI (black dashed; *P* < .001, log-rank test). AKI, acute kidney injury.

Finally, we investigated whether specific risk factors present at enrollment were associated with 28-day mortality in patients with sepsis (Supplementary Tables 8 and 9). Using 44 routinely obtained enrollment variables, such as patient characteristics, vital signs, and laboratory data (Supplementary Table 10), we performed a LASSO regression to identify a parsimonious set of variables associated with 28-day sepsis mortality. Six variables were identified, and all were associated with 28-day sepsis mortality in unadjusted models (Table 5). In a multivariable model including enrollment site, 5 risk factors were associated with an increased risk of 28-day sepsis mortality (all *P* < .01; Table 5): a preexisting HIV diagnosis, higher heart rate, lower hemoglobin, respiratory failure, and AKI (adjusted odds ratio, 2.07; 95% CI, 1.30–3.29; *P* = .002). As 39% (239/618) of patients with sepsis had AKI at enrollment, we next looked at the outcomes of sepsis cases with and without AKI. In patients with sepsis-associated AKI (SA-AKI), 15% (35/239) required kidney replacement therapy (KRT) during their hospitalization, and 30% (71/239) died within 28 days as compared with 14% (54/375) without AKI (*P* < .001). Finally, patients with sepsis with AKI at enrollment had significantly worse survival over 28-days when compared those without AKI (*P* < .001; Figure 1).

**Table 5.** Risk Factors at Presentation for 28-Day Mortality in Patients With Sepsis (n = 614)

	Unadjusted		Adjusted ^a^	
Variable ^b^	OR (95% CI)	*P* Value	OR (95% CI)	*P* Value
Preexisting conditions				
HIV	4.06 (1.28–12.8)	.02	5.28 (1.82–15.3)	.002
Presenting clinical/laboratory data				
Heart rate	1.03 (1.02–1.04)	<.001	1.03 (1.02–1.04)	<.001
Blood urea nitrogen	3.87 (1.99–7.51)	<.001	2.21 (.98–4.97)	.06
Hemoglobin	0.90 (.83–.97)	<.001	0.83 (.76–.92)	<.001
Presenting characteristics				
Acute kidney injury	2.51 (1.68–3.75)	<.001	2.07 (1.30–3.29)	.002
Respiratory failure	3.64 (2.35–5.64)	<.001	2.34 (1.42–3.86)	.001

Abbreviations: LASSO, least absolute shrinkage and selection operator; OR, odds ratio.

^a^All listed variables were included in the multivariable model, which adjusted for enrollment site (adjusted OR, 1.49; 95% CI, .94–2.37; *P* = .09).

^b^Variables included in unadjusted models and the multivariable model were selected through LASSO regression for 44 variables of patient characteristics. The sample (n = 614) does not include 4 patients lost to follow-up at 28 days after enrollment.

## DISCUSSION

This observational prospective cohort study is one of the largest multicenter studies of patients hospitalized with suspected community-acquired infection in a resource-limited setting, including Southeast Asia. We report that sepsis is common in this cohort, is caused by different pathogens as compared with high-income country (HIC) settings, and is associated with significant mortality. Furthermore, we report that critical illness is not only common but also frequently managed outside a traditional ICU. Finally, we identify SA-AKI as a significant risk factor for sepsis mortality.

Limited prospective data exist regarding the frequency of sepsis among patients hospitalized for community-acquired infection in resource-limited settings. Previously reported large prospective studies in Southeast and South Asia enrolled patients with suspected or confirmed sepsis, often only in ICUs, limiting broader application in the region [2, 5, 8, 17]. In HIC settings, 25% to 44% of patients presenting to emergency departments with community-acquired infection typically meet criteria for sepsis [18, 19]. However, in our study, 66% of patients hospitalized with community-acquired infection met sepsis criteria. Notably, patients in this study frequently received mechanical ventilation and vasoactive medications outside the ICU. These findings confirm the frequency of sepsis in this region but also highlight the challenges of applying traditional definitions of sepsis to locations with resource limitations [20, 21]. Additionally, our findings suggest that prospective enrollment of patients with sepsis in an ICU may miss patients meeting sepsis criteria and those with critical illness in similar settings. Whether patients outside the ICU requiring mechanical ventilation or vasoactive medications may benefit from an early escalation of care is unknown, although only 4% of those initially admitted to the ward with sepsis were eventually transferred to the ICU, despite a 15% mortality rate. In the United States, admission to the ward rather than the ICU may be associated with improved outcomes among patients with sepsis not requiring life support [22]. However, the 2021 SSC guidelines recommend ICU admission within 6 hours for patients requiring critical care, and early ICU transfer is associated with improved mortality in sepsis [16, 23]. In resource-limited settings where ICU transfer may not be possible for all patients who are critically ill, further study is necessary to determine the optimal management strategy and outcome effect of critical care outside the ICU.

According to the 2021 SSC guidelines, early empiric broad-spectrum antibiotics and blood cultures are recommended for patients with community-acquired sepsis [16]. Sepsis bundles have been widely implemented in Thailand, although usage varies countrywide [24]. Reflecting this, nearly all patients in our cohort received empiric antibiotics and had blood cultures sent at admission. Conversely, lactate measurement was less common, occurring in less than half of patients with sepsis. Lactate levels are associated with sepsis mortality, including that in northeast Thailand, and its measurement is recommended in the SSC hour 1 sepsis bundle [16, 25]. A recent multinational study of patients with sepsis admitted to tertiary ICUs throughout Asia reported poor compliance with 1- and 3-hour sepsis bundles [17]. However, the utility of lactate levels to guide resuscitation in patients with sepsis is unknown in LMICs and remains a topic of debate in HICs [26, 27]. Therefore, further study is necessary to determine whether broader adoption of SSC bundles is cost-efficient and improves outcomes in similar settings.

The adoption of broad, early empiric antibiotics in patients with suspected infection may contribute to antibiotic resistance, a growing concern in LMICs, including Thailand [28, 29]. Indeed, in northeast Thailand, antimicrobial-resistant infections, such as *Escherichia coli*, *Klebsiella* species, and *Acinetobacter* species, are associated with excess mortality in hospitalized patients [30]. Given this concern, the necessity of broad empiric gram-negative antibiotics in hospitalized patients in HICs has become a matter of debate [31]. In our cohort, nearly all patients, regardless of illness severity or sepsis status, received broad gram-negative coverage at admission, most commonly with ceftazidime, a third-generation cephalosporin with antipseudomonal activity, similar to prior reports in the region [32]. This approach may reflect the high frequency of gram-negative bacteremia in our cohort, a trend noted across Southeast and South Asia but distinct from many HICs [2, 5, 8, 33]. Empiric antibiotics were not discontinued quickly, as the median antibiotic duration was 6 days in patients with sepsis and 4 days in those without sepsis. Therefore, it is possible that in some patients, particularly those without sepsis and negative cultures, empiric antibiotics could have been avoided or coverage de-escalated quickly. As sepsis management in tropical settings continues to evolve, the evolution of antimicrobial stewardship programs, including biomarker-based or other de-escalation protocols, may be a reasonable approach to help address the potential for future antimicrobial resistance [7, 34].

Reflecting the increasing prevalence of diabetes mellitus in Asia, 42% of enrolled patients in this cohort had a preexisting diagnosis of diabetes [35]. The high prevalence of diabetes among patients with sepsis has been described in similar large sepsis studies in the region [2, 5, 8, 17]. In Thailand, diabetes associated with poor glycemic control is particularly prevalent in the northeast region of the country [36]. Additionally, diabetes is a major risk factor for melioidosis, perhaps reflecting the high rates of that disease in our cohort [37]. As diabetes prevalence increases in lower-resourced tropical settings, particular attention needs to be paid to how this demographic change may affect infection etiologies and management.

An intriguing finding in our study relates to the frequency and importance of SA-AKI. AKI at enrollment was common in patients with sepsis and independently associated with 28-day mortality. The burden of AKI is particularly high in LMICs, where an estimated 85% of global cases occur [38]. However, access to KRT is often limited in these settings, and continuous KRT is frequently restricted to urban quaternary facilities [39]. Only limited intermittent hemodialysis was available at the study hospitals in our report, although 15% of patients with SA-AKI at enrollment received this intervention during their hospitalization. The incidence of KRT in SA-AKI has frequently been reported as >20%, so it is possible that KRT usage would have been higher in our cohort with greater access [39, 40]. Notably, some tropical infections, including those common in rural Southeast Asia, such as leptospirosis and melioidosis, are associated with particularly increased risks of AKI [41, 42]. While it is unclear whether SA-AKI is preventable, optimized management may reduce the risk of disease progression [43]. Additionally, recent reports have identified subphenotypes of HIC AKI cases with potentially unique responses to therapeutic interventions [44]. Critically, few studies have assessed the epidemiology of SA-AKI in resource-constrained settings globally, including Southeast Asia [45]. Given the prevalence and importance of this sepsis-associated disease, further contextualized consideration of SA-AKI in global settings is urgently needed.

This study has several strengths. To our knowledge, this is the largest multicenter prospective cohort study of patients hospitalized with community-acquired infection in a resource-limited setting. Our study included all-cause 28-day mortality, important given the local culture practice of discharging moribund patients to die at home, with minimal loss to follow-up. Study data were collected by a trained study team, yielding minimal missing data. Additionally, strict, broadly accepted definitions were used to define patients with sepsis and AKI.

Our study also has several limitations. Highlighting the resource constraints of the study hospitals, arterial blood gases were not routinely obtained, requiring modification of SOFA. Additionally, the timing of specific interventions, including antibiotics, was not available on a minute or hour level of specificity, rather the calendar day. Identification of infectious etiologies was limited to the testing performed at the study hospitals and did not include prospective specific pathogen testing. Antibiotic susceptibility data were not available either. Both study hospitals were referral hospitals within their rural provinces; therefore, care delivery may have differed among hospitals and may have been affected by the prospective study design. Additionally, patient demographics and management may differ as compared with local health care centers or larger academic quaternary care centers within Thailand and other global settings. The study was also conducted over a 16-month period and may not completely reflect the seasonal variations of some infections; moreover, it did not enroll patients infected with SAR-CoV-2.

In conclusion, sepsis and sepsis-associated critical illness are common and associated with mortality in adults with community-acquired infection in rural Southeast Asia. SA-AKI on presentation was associated with an increased risk of mortality. Given the paucity of high-quality existing data on sepsis in resource-constrained settings, this study provides critical and timely data to inform future clinical and research approaches in global settings.

## Supplementary Material

ofag022_Supplementary_Data

## Supplementary Data

Supplementary materials are available at *Open Forum Infectious Diseases* online. Consisting of data provided by the authors to benefit the reader, the posted materials are not copyedited and are the sole responsibility of the authors, so questions or comments should be addressed to the corresponding author.
