# Supplementary material for: Epidemiology, Management, and Outcomes of Patients Hospitalized With Community-Acquired Infection in a Resource-Limited Setting in Southeast Asia: A Prospective Observational Study
Source: Open Forum Infect Dis. 2026 Jan 14;13(2):ofag022. doi: 10.1093/ofid/ofag022 (PMC12866911; doi:10.1093/ofid/ofag022)
Supplement: ofag022_Supplementary_Data [file ofag022_supplementary_data.pdf]

Online Supplement to: Epidemiology, management, and outcomes of patients hospitalized with community-acquired infection in a resource-limited setting in Southeast Asia: a prospective observational study.

Supplementary information

Contents (Page):

Supplemental Figure 1: Study flow diagram (2)
Supplemental Table 1: Respiratory SOFA score modifications (3)
Supplemental Table 2: Data availability (4)
Supplemental Table 3: Site characteristics of patients with community-acquired infection (5)
Supplemental Table 4: Site management, severity of illness and outcomes of patients (6)
Supplemental Table 5: Antibiotic management in patients with community-acquired infection (7)
Supplemental Table 6: Infectious etiologies of patients with community-acquired infection (8)
Supplemental Table 7: Characteristics of patients with sepsis and critical illness inside and outside the ICU (9)
Supplemental Table 8: Characteristics of patients with sepsis by survival (10)
Supplemental Table 9: Infectious etiology and management in patients with sepsis by survival (11)
Supplemental Table 10: Patient characteristics association with sepsis-related 28-day (12-13)
Supplemental Methods & References (14-16)

Supplemental Figure 1: Study flow diagram

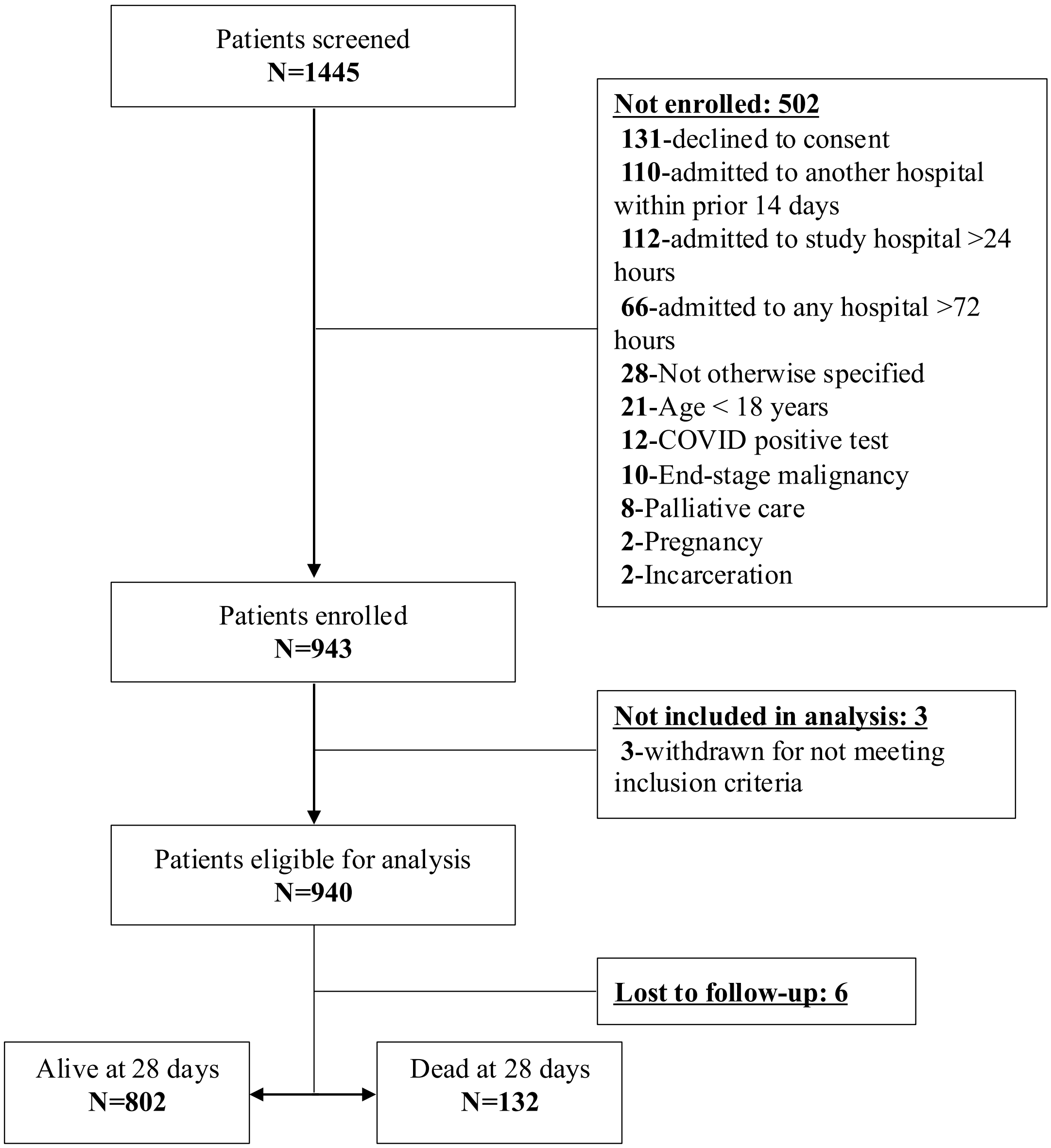

Supplemental Table 1: Respiratory SOFA score modifications

Score	PaO_2/FiO_2	PaO_2/FiO_2 calculated from SpO_2/FiO_2 if SpO_2<98%	PaO_2/FiO_2 unavailable & $SpO_2 \geq 98\%$
**0**	≥400	≥400	no mechanical ventilation
**1**	<400	<400	
**2**	<300	<315	mechanical ventilation
**3**	<200 + mechanical ventilation	<232 + mechanical ventilation	
**4**	<100 + mechanical ventilation	<148 + mechanical ventilation	

[1] Respiratory SOFA scores calculated preferentially using PaO_2/FiO_2. If PaO_2/FiO_2 not available, a calculated PaO_2/FiO_2 was used if SpO_2<98%. If SpO_2>98% and no PaO_2/FiO_2 was available, then presence of mechanical ventilation was used for the respiratory SOFA score. Patients not meeting these parameters were given a zero.

Supplemental Table 2: Data availability

Variable, total available (%)	Entire cohort (N=940)
**Baseline risk factors**	
Sex	940 (100)
Age	940 (100)
Pre-existing condition data	940 (100)
Referral status	940 (100)
**Enrollment modified SOFA**	
Mechanical ventilation	940 (100)
PaO_2	239 (25)
SpO_2	904 (96)
Platelet count	938 (99)
Total bilirubin	642 (68)
Mean arterial blood pressure	940 (100)
Vasoactive medications	940 (100)
Glasgow Coma Scale	940 (100)
Creatinine	939 (99)
**Outcome at 28 days**	934 (99)

Supplemental Table 3: Site characteristics of patients with community-acquired infection

Characteristics	Entire cohort (N=940)	Mukdahan Hospital (N=593)	Roi Et Hospital (N=347)	P-value
**Demographics**				
Age in years, median (IQR)	60 (49-70)	58 (48-68)	64 (50-74)	<0.001
Female sex, N (%)	389 (41)	345 (58)	206 (59)	0.72
Rainy season presentation, N (%)	614 (65)	417 (70)	197 (57)	<0.001
**Pre-existing conditions**				
Charlson Comorbidity Index, median (IQR)	2 (1-4)	2 (1-4)	3 (1-4)	0.002
Diabetes, N (%)	396 (42)	286 (48)	110 (32)	<0.001
Hypertension, N (%)	356 (38)	209 (35)	147 (42)	0.03
Chronic kidney disease, N (%)	139 (15)	85 (14)	54 (16)	0.60
Dyslipidemia, N (%)	97 (10)	65 (11)	32 (9)	0.40
Stroke, N (%)	60 (6)	28 (5)	32 (9)	<0.01
Chronic lung disease, N (%)	55 (6)	27 (5)	28 (8)	0.03
Chronic cardiovascular disease, N (%)	41 (4)	16 (3)	25 (7)	0.001
Chronic steroid use, N (%)	34 (4)	20 (3)	14 (4)	0.59
Cancer, N (%)	24 (3)	11 (2)	13 (4)	0.08
Chronic liver disease, N (%)	24 (3)	12 (2)	12 (3)	0.18
HIV, N (%)	17 (2)	9 (2)	8 (2)	0.45
Rheumatologic disorders, N (%)	16 (2)	8 (1)	8 (2)	0.30
**Hospitalization**				
Referral from another facility	416 (44)	170 (29)	246 (71)	<0.001
Days to referral, median (IQR)	0 (0-0)	0 (0-0)	0 (0-0)	0.61
Admission ward				<0.001
Medical	810 (86)	489 (83)	321 (92)	
Surgical	28 (3)	28 (5)	0	
ICU	102 (11)	76 (13)	26 (8)	
Length of hospitalization (days)	6 (3-13)	6 (3-13)	5 (3-10)	<0.001
Procedural drainage/debridement	87 (9)	54 (9)	33 (10)	0.84
**Presenting clinical syndromes**[2]				
Pneumonia, N (%)	354 (38)	160 (27)	194 (56)	<0.001
Acute febrile illness, N (%)	204 (22)	140 (24)	64 (18)	0.06
Gastrointestinal illness, N (%)	132 (14)	92 (16)	40 (12)	0.09
Skin or soft tissue infection, N (%)	27 (3)	16 (3)	11 (3)	0.68
Urinary tract infection, N (%)	73 (8)	33 (6)	40 (12)	0.001
Intracranial infection, N (%)	4 (0)	3 (1)	1 (0)	1.0
Abscess, N (%)	33 (4)	21 (4)	12 (3)	0.95

[1] Presenting clinical syndromes based on primary admission diagnosis

Supplemental Table 4: Site management, severity of illness and outcomes of patients

Characteristics	Entire cohort (N=940)	Mukdahan Hospital (N=593)	Roi Et Hospital (N=347)	P-value
**Admission management**				
Blood cultures sent on admission	902 (96)	585 (99)	317 (91)	<0.001
Lactate measured on admission	302 (32)	61 (10)	211 (77)	<0.001
**Admission illness characteristics**				
SOFA, median (IQR)	3 (1-7)	2 (0-6)	5 (2-8)	<0.001
qSOFA, median (IQR)	1 (1-2)	1 (1-2)	2 (1-2)	<0.001
Acute kidney injury[1], N (%)	261 (28)	156 (26)	105 (30)	0.19
**Critical care at enrollment**				
Respiratory failure[2], N (%)	306 (33)	119 (20)	187 (54)	<0.001
Mechanical ventilation, N (%)	277 (29)	110 (19)	167 (48)	<0.001
Vasoactive medications, N (%)	198 (21)	125 (21)	73 (21)	0.99
New kidney replacement therapy[3], N (%)	15 (2)	14 (2)	1 (0)	0.01
**Outcomes**				
Death				
7 days, N (%)	78 (8)	36 (6)	42 (12)	0.001
Days to death, median (IQR)	2 (1-5)	1 (1-4)	3 (1-6)	0.02
28 days, N (%)	132 (14)	61 (10)	71 (21)	<0.001
Days to death, median (IQR)	6 (2-13)	5 (1-12)	6 (2-16)	0.29
Lost to 28-day follow-up, N (%)	6 (1)	3 (1)	3 (1)	0.68

[1]Acute kidney injury based on KDIGO definition of 150% of estimated baseline creatinine in patients without chronic kidney disease or increase in creatinine by 0.3 mg/dL within 48 hours in any patient.
[2] Respiratory failure defined as requiring either endotracheal intubation, mechanical ventilation or non-invasive positive pressure ventilation.
[3] Kidney replacement therapy includes new receipt of hemodialysis or peritoneal dialysis.

Supplemental Table 5: Antibiotic management in patients with community-acquired infection

Characteristics	Entire cohort (N=940)	Mukdahan Hospital (N=593)	Roi Et Hospital (N=347)
**Antibiotics received on admission**	917 (98)	582 (98)	335 (97)
Broad gram-negative coverage[1]	912 (97)	579 (98)	333 (96)
MRSA coverage[2]	185 (20)	116 (20)	69 (20)
Duration, median days (IQR)	6 (3-12)	6 (3-13)	4 (2-8)
**Admission antibiotics at study hospitals[3]**			
Ceftazidime	607 (65)	495 (84)	112 (32)
Ceftriaxone	294 (31)	92 (16)	202 (58)
Clindamycin	87 (9)	63 (11)	24 (7)
Doxycycline	78 (8)	47 (8)	31 (9)
Meropenem	49 (5)	18 (3)	31 (9)
Piperacillin-tazobactam	45 (5)	13 (2)	32 (9)
**Antibiotics received at referring sites[4]**		**Referred**	
	**(N=416)**	**(N=170)**	**(N=246)**
Ceftazidime	161 (39)	89 (52)	72 (29)
Ceftriaxone	217 (52)	59 (35)	158 (64)
Clindamycin	26 (6)	11 (7)	15 (6)
Doxycycline	22 (5)	9 (5)	13 (5)
Meropenem	12 (3)	1 (0)	11 (4)
Piperacillin-tazobactam	6 (1)	0	6 (2)

[1] Gram-negative coverage on or prior to admission include receipt of a third- or fourth-generation cephalosporin, carbapenem, piperacillin-tazobactam, ampicillin-sulbactam or levofloxacin.
[2] Community-acquired methicillin resistant *Staphylococcus aureus* coverage on or prior to admission include receipt of vancomycin, doxycycline, or clindamycin.
[3] Antibiotics received at study hospitals at the time of admission. Multiple antibiotics could be received concurrently; six most commonly received antibiotics listed.
[4] Antibiotics received at referring hospitals prior to transfer. Multiple antibiotics could be received concurrently; six most commonly received antibiotics listed. Percentages refer to total referred, including to each site.

Supplemental Table 6: Infectious etiologies of patients with community-acquired infection

Characteristics	Entire cohort (N=940)	Mukdahan Hospital (N=593)	Roi Et Hospital (N=347)	P-value
**Blood stream infection**	164 (18)	105 (18)	59 (17)	0.78
Gram-negative bacteremia	133 (14)	87 (15)	46 (13)	0.55
Burkholderia pseudomallei	58 (6)	44 (8)	14 (4)	0.04
Escherichia coli	39 (4)	22 (4)	17 (5)	0.38
Klebsiella spp.	21 (2)	15 (3)	6 (2)	0.50
Pseudomonas spp.	3 (0)	2 (0)	1 (0)	1.0
Acinetobacter baumannii	5 (1)	1 (0)	4 (1)	0.07
Salmonella serogroup D	5 (1)	0	5 (1)	<0.01
Citrobacter koseri	2 (0)	2 (0)	0	0.53
Vibrio parahemolyticus	1 (0)	1 (0)	0	1.0
Aeromonas spp.	1 (0)	1 (0)	0	1.0
Gram-positive bacteremia	32 (3)	18 (3)	14 (4)	0.42
Staphylococcus aureus	11 (1)	5 (1)	6 (2)	0.23
Streptococcus pneumoniae	4 (0)	2 (0)	2 (1)	0.63
Streptococcus pyogenes	6 (1)	6 (1)	0	0.09
Group G *Streptococcus*	3 (0)	2 (0)	1 (0)	1.0
Group D *Streptococcus*	2 (0)	1 (0)	1 (0)	1.0
Other *Streptococcus* spp.	2 (0)	1 (0)	1 (0)	1.0
Enterococcus faecalis	4 (0)	1 (0)	3 (1)	0.15
Polymicrobial	5 (1)	2 (0)	3 (1)	0.36
Contaminant organisms[1]	35 (4)	13 (2)	22 (6)	<0.01
**Other infectious diseases[2]**				
Leptospirosis	36 (4)	30 (5)	6 (2)	0.01
Acute tuberculosis disease	24 (3)	13 (2)	11 (3)	0.36
Non-bacteremic melioidosis	20 (2)	18 (3)	2 (1)	<0.01
Scrub typhus	14 (2)	9 (2)	5 (1)	1.0
Dengue	7 (1)	5 (1)	2 (1)	1.0
Malaria	1 (0)	0	1 (0)	0.37

[1] Contaminant organisms include coagulase-negative *Staphylococcus* spp, alpha-hemolytic *Streptococcus* spp, *Propionibacterium* spp, *Corynebacterium* spp, *Burkholderia cepacia*, or *Bacillus* spp if no other clinical evidence existed suggestive of infection.

[2] Listed infectious etiologies were made based on final diagnoses using local hospital testing and diagnostic protocols. Non-bacteremic melioidosis diagnoses were made based on positivity of any non-blood culture for *B. pseudomallei*.

Supplemental Table 7: Characteristics of patients with sepsis and critical illness inside and outside the ICU

Characteristics	ICU (N=174)	Ward[1] (N=213)	P-value
**Severity of illness at presentation**			
SOFA, median (IQR)	10 (7-13)	6 (4-9)	<0.001
APACHE II, median (IQR)	22 (17-25)	16 (13-21)	<0.001
Acute kidney injury[2], N (%)	94 (54)	81 (38)	0.002
**Critical care at enrollment**			
Respiratory failure, N (%)	159 (91)	147 (69)	<0.001
Mechanical ventilation, N (%)	156 (90)	131 (62)	<0.001
Vasoactive medications, N (%)	106 (61)	92 (43)	0.001
New kidney replacement therapy, N (%)	32 (18)	8 (4)	<0.001
**Management**			
Blood cultures sent on admission	172 (99)	203 (95)	0.07
Antibiotics received on admission	172 (99)	208 (98)	0.47
Procedural drainage/debridement	16 (9)	16 (8)	0.55
Lactate measured on admission	96 (55)	119 (56)	0.89
**ICU admission for entire hospitalization**			
Any ICU admission, N (%)	174 (100)	13 (6)	<0.001
Total ICU length of stay (median days, IQR)	5 (3-10)	8 (3-14)	0.40
**Death**			
7 days, N (%)	38 (22)	27 (13)	0.02
Days to death, median (IQR)	2 (1-5)	2 (1-4)	0.82
28 days, N (%)	60 (34)	41 (19)	0.001
Days to death, median (IQR)	5 (1-12)	4 (1-9)	0.69
Lost to 28-day follow-up, N (%)	0	2 (1)	0.50

[1] Critical illness outside the ICU defined as requiring advanced respiratory support, vasoactive medications or new kidney replacement therapy at the time of enrollment.

[2] Acute kidney injury based on KDIGO definition of 150% of estimated baseline creatinine in patients without chronic kidney disease or increase in creatinine by 0.3 mg/dL within 48 hours in any patient not requiring dialysis at baseline.

Supplemental Table 8: Characteristics of patients with sepsis by survival

Characteristics	Sepsis (N=614)	28-day outcome Survived (N=489)	28-day outcome Died (N=125)	P-value
**Demographics**				
Age in years, median (IQR)	61 (50-72)	62 (50-72)	59 (49-72)	0.75
Female sex, N (%)	232 (38)	185 (38)	47 (38)	0.96
Rainy season presentation, N (%)	388 (63)	319 (65)	69 (55)	0.04
Site, N (%)				
Mukdahan Hospital	342 (56)	283 (58)	59 (47)	0.03
Roi Et Hospital	272 (44)	205 (42)	66 (53)	
**Pre-existing conditions**				
Charlson Comorbidity Index, median (IQR)	3 (1-4)	3 (1-4)	3 (1-5)	0.36
Diabetes, N (%)	243 (40)	197 (40)	46 (37)	0.48
Chronic liver disease, N (%)	24 (4)	15 (3)	9 (8)	0.03
Chronic kidney disease, N (%)	114 (19)	89 (18)	25 (20)	0.65
Chronic cardiovascular disease, N (%)	35 (6)	27 (6)	8 (7)	0.67
Chronic lung disease, N (%)	34 (6)	28 (6)	6 (5)	0.83
Cancer, N (%)	17 (3)	14 (3)	3 (2)	1.00
Stroke, N (%)	45 (7)	33 (7)	12 (10)	0.28
HIV, N (%)	12 (2)	6 (1)	6 (5)	0.02
Hypertension, N (%)	239 (39)	196 (40)	43 (34)	0.25
Dyslipidemia, N (%)	60 (10)	53 (11)	7 (6)	0.09
Rheumatologic disorders, N (%)	12 (2)	9 (2)	3 (2)	0.72
Chronic steroid use, N (%)	21 (3)	14 (3)	7 (6)	0.16
**Hospitalization**				
Referral from another facility	348 (57)	266 (54)	82 (66)	0.02
Days to referral, median (IQR)	0 (0-0)	0 (0-0)	0 (0-0)	0.59
Admission ward				
Medical	505 (82)	415 (85)	90 (72)	0.001
Surgical	9 (2)	8 (2)	1 (1)	
ICU	100 (16)	66 (14)	34 (27)	
Length of hospitalization (days)	7 (3-13)	7 (4-13)	5 (2-9)	<0.001
**Presenting clinical syndromes**[1]				
Pneumonia, N (%)	269 (44)	200 (41)	69 (55)	0.004
Acute febrile illness, N (%)	110 (18)	100 (21)	10 (8)	0.001
Gastrointestinal illness, N (%)	80 (13)	58 (12)	22 (18)	0.09
Skin or soft tissue infection, N (%)	14 (2)	9 (2)	5 (4)	0.18
Urinary tract infection, N (%)	48 (8)	43 (9)	5 (4)	0.09
Intracranial infection, N (%)	3 (1)	2 (0)	1 (1)	0.50
Abscess, N (%)	14 (2)	12 (3)	2 (2)	0.75

[1] Presenting clinical syndromes based on primary admission diagnosis.

Supplemental Table 9: Infectious etiology and management in patients with sepsis by survival

Characteristics	Sepsis (N=614)	28-day outcome Survived (N=489)	Died (N=125)	P-value
**Blood stream infection**	128 (21)	91 (19)	37 (30)	0.01
Gram-negative bacteremia	100 (16)	70 (14)	30 (24)	0.01
B. pseudomallei	43 (7)	27 (6)	16 (13)	0.004
Gram-positive bacteremia	29 (5)	21 (4)	8 (6)	0.34
**Other infectious diseases**[1]				
Non-bacteremic melioidosis	12 (2)	8 (2)	4 (4)	0.26
Dengue	4 (1)	4 (1)	0	0.59
Malaria	1 (0)	1 (0)	0	1.0
Leptospirosis	33 (5)	29 (6)	4 (3)	0.27
Acute tuberculosis disease	10 (2)	10 (2)	0	0.23
Scrub typhus	12 (2)	12 (3)	0	0.14
**Enrollment illness characteristics**				
SOFA, median (IQR)	5 (3-9)	5 (3-8)	9 (5-13)	<0.001
qSOFA, median (IQR)	2 (1-2)	2 (1-2)	2 (2-3)	<0.001
Acute kidney injury, N (%)	239 (39)	168 (34)	71 (57)	<0.001
Respiratory failure, N (%)	304 (50)	212 (43)	92 (74)	<0.001
Mechanical ventilation, N (%)	275 (45)	185 (38)	90 (72)	<0.001
Vasoactive medications, N (%)	198 (32)	143 (27)	64 (51)	<0.001
New hemodialysis, N (%)	15 (2)	14 (3)	1 (1)	0.18
**Admission management**				
Blood cultures	592 (96)	471 (96)	121 (97)	1.0
Antibiotics received	600 (98)	477 (98)	123 (98)	0.75
Broad gram-negative coverage[2]	599 (98)	477 (98)	122 (98)	1.0
MRSA coverage[3]	125 (20)	96 (20)	29 (23)	0.39
Lactate measured	261 (43)	190 (39)	71 (57)	<0.001

[1] Listed infectious diseases were made based on final diagnoses using local hospital testing and diagnostic protocols. Non-bacteremic melioidosis diagnoses were made based on positivity of any non-blood culture for *B. pseudomallei*.

[2] Gram-negative coverage on or prior to admission include receipt of a third- or fourth-generation cephalosporin, carbapenem, piperacillin-tazobactam, ampicillin-sulbactam or levofloxacin.

[3] Community-acquired MRSA coverage on or prior to admission include receipt of vancomycin, doxycycline or clindamycin.

Supplemental Table 10: Patient characteristics and sepsis-related 28-day mortality

Variable	Unadjusted OR	95% CI
**Demographics**		
Age	1.00	0.99-1.01
Sex	1.01	0.67-1.52
Enrollment site	1.53	1.04-2.28
**Pre-existing conditions**		
Charlson Comorbidity Index	1.08	0.98-1.20
Diabetes mellitus	0.86	0.57-1.30
Hypertension	0.78	0.52-1.18
Chronic kidney disease	1.12	0.68-1.84
Dyslipidemia	0.49	0.22-1.10
Stroke	1.47	0.73-2.93
Chronic lung disease	0.83	0.34-2.05
Chronic cardiovascular disease	1.18	0.52-2.64
Cancer	0.83	0.24-2.95
Chronic liver disease	2.45	1.05-5.75
HIV	4.06	1.29-12.8
Rheumatologic disorders	1.31	0.35-4.92
**Hospitalization**		
Referral from another facility	1.60	1.06-2.41
**Presenting clinical syndromes**		
Pneumonia	1.78	1.20-2.65
Acute febrile illness	0.34	0.17-0.67
Gastrointestinal illness	1.59	0.93-2.71
Skin or soft tissue infection	2.22	0.73-6.76
Urinary tract infection	0.43	0.17-1.12
Intracranial infection	1.96	0.18-21.9
Abscess	0.64	0.14-2.93
**Presenting laboratory data**		
Blood glucose	0.44	0.16-1.23
Blood urea nitrogen	3.87	1.99-7.52
Creatinine	1.95	1.17-3.25
Bicarbonate	0.12	0.04-0.37
Sodium	0.03	$2.4E^{-6}$-491
White blood cell count	0.56	0.27-1.15
Neutrophil count	0.55	0.30-1.01
Lymphocyte count	0.51	0.31-0.84
Monocyte count	0.67	0.39-1.16
Neutrophil:lymphocyte ratio	1.19	0.73-1.94
Hemoglobin	0.90	0.83-0.97
Platelet count	0.63	0.36-1.09

**Presenting clinical data**		
Receiving vasoactive medications	2.78	1.86-4.16
Respiratory failure	3.64	2.35-5.64
Acute kidney injury	2.51	1.68-3.75
Body temperature	1.00	0.83-1.20
Respiratory rate	1.05	1.02-1.08
Heart rate	1.03	1.02-1.04
Systolic blood pressure	0.99	0.98-1.00
Diastolic blood pressure	0.98	0.97-1.00
Mean arterial blood pressure	0.98	0.97-1.00

Supplemental Methods

Cohort exclusion criteria

Exclusion criteria included admission to the study hospital for > 24 hours prior to enrollment, admission to any hospital (including referring hospitals) > 72 hours prior to enrollment, hospital admission within the prior 14 days, end-stage oncologic malignancies, palliative care, pregnancy or incarceration. In addition, patients were excluded if they had a positive COVID-19 PCR or antigen test within 14 days of admission.

Study design

Trained study personnel sequentially screened admission logs and medical records of patients in the emergency room, intensive care units and medical wards. Medical staff also alerted study personnel of potentially eligible patients. After informed consent was obtained, clinical data was extracted from the medical chart or from the patient or surrogate decision-maker using a standardized case report form.

Site and regional information

Patients were enrolled at Mukdahan Hospital (Mukdahan Province) and Roi Et Hospital (Roi Et Province), both located in the northeast region of Thailand. This northeast region is the poorest in Thailand and, per the World Bank, is a rural, agrarian economy which has experienced less economic growth than other regional neighbors in the Greater Mekong Subregion, including Laos and Cambodia [1]. In addition to the high poverty rate, the northeast region is characterized

by Thailand's poorest health outcomes and highest socioeconomic inequalities and unmet healthcare needs [2–4]. Mukdahan Hospital is a 485-bed hospital with 8 medical ICU beds located in Mukdahan Province. Roi Et hospital is an 881-bed hospital with 34 medical ICU beds.

Definitions

Presenting clinical syndromes were classified based on the primary diagnosis listed by the admitting physician. For patients admitted to the ICU, an APACHE II score was calculated using data available at the time of enrollment [5]. All culture results available during hospitalization were obtained from the respective hospital microbiology labs. Bacteremia was defined as a positive blood culture obtained within 24 hours of hospital admission. Blood cultures positive for organisms typically associated with contamination were not classified as bacteremia, including coagulase-negative staphylococci, alpha-hemolytic streptococci, *Micrococcus* spp., *Diphtheroid* spp., *Propionibacterium* spp., *Corynebacterium* spp. or *Bacillus* spp. A positive culture of *Burkholderia pseudomallei* from any sample type was considered consistent with melioidosis. Diagnoses of other infectious etiologies, including malaria, leptospirosis, dengue fever, acute tuberculosis disease and scrub typhus were made based on a combination of hospital laboratory testing and the final diagnosis at discharge.

Supplemental References

1. World Bank and National Economic and Social Development Board. Thailand Northeast Economic Development Report. 2005.
2. Kaikeaw S, Punpuing S, Chamchan C, Prasartkul P. Socioeconomic inequalities in health outcomes among Thai older population in the era of Universal Health Coverage: trends and decomposition analysis. Int J Equity Health 2023; 22:144.
3. Sritart H, Tuntiwong K, Miyazaki H, Taertulakarn S. Disparities in healthcare services and spatial assessments of mobile health clinics in the border regions of Thailand. Int J Environ Res Public Health 2021; 18:10782.
4. Chongthawonsatid S. Identification of unmet healthcare needs: A national survey in thailand. Journal of Preventive Medicine and Public Health 2021; 54:129–136.
5. Knaus WA, Draper EA, Wagner DP, Zimmerman JE. APACHE II: a severity of disease classification system. Crit Care Med 1985; 13:818–829.
